# The Role of MicroRNA in Contrast-Induced Nephropathy: A Scoping Review and Meta-Analysis

**DOI:** 10.1155/2020/4189621

**Published:** 2020-05-21

**Authors:** Mangiring P. L. Toruan, Raymond Pranata, Budi Yuli Setianto, Sofia Mubarika Haryana

**Affiliations:** ^1^Department of Cardiology and Vascular Medicine, Mohammad Hoesin General Hospital, Palembang, Sumatera Selatan, Indonesia; ^2^Biomedicine Doctoral Programme, Faculty of Medicine, Public Health, and Nursing, Universitas Gadjah Mada/Sardjito General Hospital, Yogyakarta, Indonesia; ^3^Faculty of Medicine, Universitas Pelita Harapan, Tangerang, Indonesia; ^4^Department of Cardiology and Vascular Medicine, Faculty of Medicine, Public Health, and Nursing, Universitas Gadjah Mada/Sardjito General Hospital, Yogyakarta, Indonesia; ^5^Histology and Cell Biology Department, Faculty of Medicine, Public Health, and Nursing, Gadjah Mada University, Yogyakarta, Indonesia

## Abstract

**Objective:**

Early diagnosis of contrast-induced nephropathy (CIN) remains crucial for successful treatment; unfortunately, the widely used serum creatinine is elevated only in the late stage of CIN. The circulating microRNAs (miRNAs) are stable biomarker that might be useful. The aim of this scoping review and meta-analysis is to assess the role of miRNAs in CIN.

**Methods:**

We performed a systematic literature search on topics that assess the role of miRNAs in CIN from several electronic databases.

**Results:**

There were 6 preclinical studies and 2 of them validated their findings in human. Only miR-30a, miR-30c, miR-30e, and miR-188 have been validated in human models. Meta-analysis showed that increase in miR-30a expression was associated with higher incidence of CIN (OR 4.48 [1.52, 13.26],  *p* = 0.007; *I*^2^: 94%, *p* < 0.001). An increase in miR-30e expression was associated with higher incidence of CIN (OR 2.34 [1.70, 3.20], *p* < 0.001; *I*^2^: 0%, *p* = 0.76). There is an indication that miR-188 is associated with contrast-induced apoptosis and might potentially be a drug target in the future.

**Conclusion:**

This study highlighted the importance of certain miRNAs in CIN pathophysiology. Future researches should explore on the prognostic and therapeutic implication of miRNA in CIN.

## 1. Introduction

The incidence of contrast-induced nephropathy (CIN) is estimated to be >2% in the general population; however, it may rise up to >30% in high-risk patients [1]. CIN increases morbidity and mortality of patients undergoing coronary angiography (CAG) and percutaneous coronary intervention (PCI) [2]. There are various definition for CIN, one of the most popular is the increase in serum creatinine level ≥ 0.5 mg/dL (44.2 mmol/L) or >25% of the baseline value 48–72 h following the administration of contrast media (CM) [3].

Early diagnosis of CIN remains crucial in order for a treatment to be successful; unfortunately, the widely used serum creatinine is elevated only in the late stage of CIN [4]. MicroRNAs (miRNAs) are posttranscriptional regulators that binds to target mRNAs and degrade them [5]. The circulating miRNAs are stable biomarker that might be useful[6, 7]; they also regulate and reflect disease-specific molecular pathways, allowing a more precise discrimination of the pathology [8]. Alteration in miRNA expression has been demonstrated in both acute and chronic kidney diseases [5]. The crucial role of miRNA in the regulation of cell functions and disease-specific molecular pathways allow a more precise molecular drug targeting [9]. The first study of miRNA assessment in CIN was conducted in 2015; the authors of the study reported that miRNAs might serve as an early biomarker and target candidates for CIN [4]. The aim of this scoping review and meta-analysis is to assess the role of miRNAs in CIN.

## 2. Methods

### 2.1. Search Strategy

**Figure 1:**
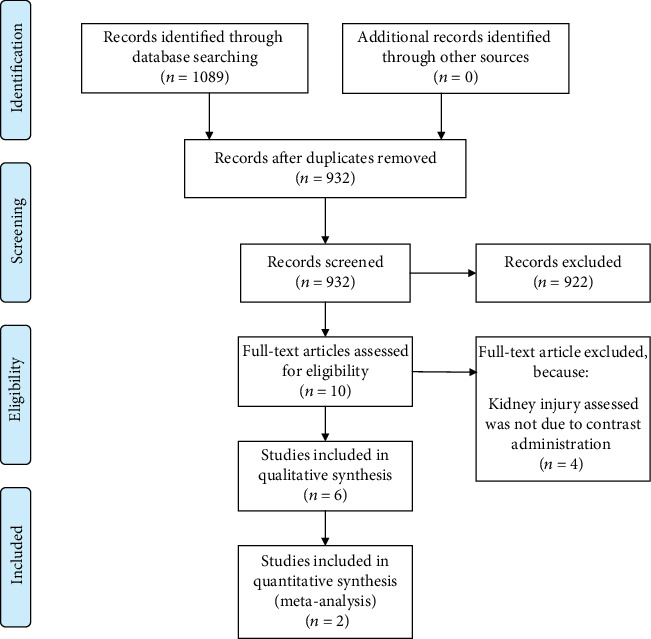
Study flow diagram.

We performed a systematic literature search on studies that assess the role of miRNAs in CIN with keywords [“microrna” and “contrast induced nephropathy”], including its synonym from inception until December 2019 from several electronic literature databases including PubMed, EuropePMC, Cochrane Central Database, ScienceDirect (research articles), ProQuest, ClinicalTrials.gov, and hand sampling/snowballing from potential articles cited by other studies. After initial search, the title/abstract of the records were then systematically evaluated by applying inclusion and exclusion criteria. Two independent researchers performed the initial search; discrepancies that arose were resolved by discussion. A Preferred Reporting Items for Systematic Reviews and Meta-Analyses (PRISMA) flowchart of the literature search is available as Figure 1.

### 2.2. Selection Criteria

The inclusion criteria for this study was all studies that assessed the role of miRNAs in CIN. We included related original articles including clinical researches and animal studies. We excluded case reports, review articles, and non-English language articles.

### 2.3. Data Extraction

Data extraction and quality assessment were performed by two independent authors using a standardized extraction form which include authors, year of publication, the animal model used, whether human validation was performed, contrast used, control group, CIN definition, and whether miRNA was validated.

### 2.4. Statistical Analysis

To perform the meta-analysis, we used RevMan version 5.3 software (Cochrane Collaboration). We pooled odds ratio (OR) using generic inverse variance with the random-effects model regardless of heterogeneity to generate pooled effect estimate and its 95% confidence interval. To assess heterogeneity, we used the inconsistency index (*I*^2^) test, which ranges from 0 to 100%. A value above 50% or *p* < 0.10 indicates a statistically significant heterogeneity. All *p* values of the effect estimate were two-tailed with a statistical significance set at 0.05 or below. Assessment for publication bias was not performed due to the lack of studies.

## 3. Results

### 3.1. Study Selection and Characteristics

Initial search yielded 1089 results, in which 932 records remained after the removal of duplicates. 922 records that did not satisfy inclusion/criteria criteria were excluded after the screening of title/abstracts. The residual 10 full-text articles were then assessed for eligibility, we excluded 4 because the etiology of kidney injury assessed was not due to contrast administration. We included 6 studies in qualitative synthesis and 2 in meta-analysis [4, 10–14] (Figure 1). There are 6 preclinical studies and 2 of them validated their findings in humans. The studies used varying types of contrast agents. The definition of CIN used by the studies was an absolute increase of serum creatinine ≥0.3-0.5 mg/dL or a relative increase of >25% from the baseline value in 24-48/48-72 hours after CM administration. Animal models underwent dehydration protocol to induce CIN. Sun et al. and Gutierrez-Escolano et al. performed validation in a human model (Table 1).

### 3.2. MicroRNA Upregulation and Contrast-Induced Nephropathy

In an animal study by Gutierrez-Escolano et al., there were 17 miRNAs that have been shown to increase by twofold in the CIN group compared to control group. Among the 17 aberrantly expressed miRNAs, only 6 of them showed significantly different expression in the plasma of CIN rats. miR-30a, miR-30c, miR-30e, and miR-320 were significantly increased in CIN rats. Sun et al. study showed that miR-188, miR-30a, and miR-30e were higher in the CIN rats. Liu et al. demonstrated that the miR-188 was significantly upregulated in the rat and HK-2 cell model with CIN. In Liu et al. study, a novel rat model with nonionic low-osmolar iodic contrast medium iopromide was used. 19 miRNAs were shown to be upregulated, in which miR-3558-5p, miR-34c-3p, and miR-384-5p were the most significantly elevated. Wang et al. study reported 23 upregulated miRNAs, and the most upregulated were miR-122-5p, miR-126a-5p, and miR-376b-3p. Cheng et al. reported 16 upregulated miRNAs, and miR-201-3p was the most upregulated. The miRNAs that have been validated in humans were miR-30a, miR-30c, miR-30e, and miR-188.

### 3.3. MicroRNA Downregulation and Contrast-Induced Nephropathy

Gutierrez-Escolano et al. reported that miRNAs let-7a and miR-200a were lower in CIN rats. Liu et al. showed that miR-328a-5p, miR-31a-5p, and miR-377-3p were the most significantly downregulated miRNAs out of 22. miR-1298, miR-378b, and miR-362-5p were reported to be the most downregulated miRNAs in Wang et al. study. Cheng et al. reported 22 downregulated miRNAs, and miR-144-3p was the most downregulated.

### 3.4. Meta-Analysis of MicroRNA and Incidence of Contrast-Induced Nephropathy

**Figure 2:**
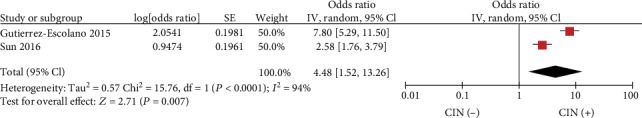
MicroRNA-30a and contrast-induced nephropathy. Upregulated microRNA-30a was shown to be associated with CIN. CIN: Contrast-induced nephropathy.

**Figure 3:**
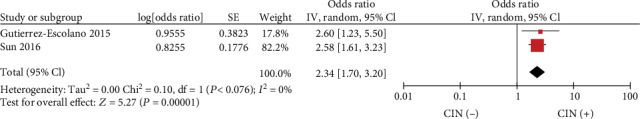
MicroRNA-30e and contrast-induced nephropathy. Upregulated microRNA-30e was shown to be associated with CIN. CIN: Contrast-induced nephropathy.

Only data regarding miR-30a and miR-30e can be meta-analyzed. Meta-analysis showed that an increase in miR-30a expression was associated with higher incidence of CIN (OR 4.48 [1.52, 13.26], *p* = 0.007; *I*^2^: 94%, *p* < 0.001) (Figure 2). An increase in miR-30e expression was associated with higher incidence of CIN (OR 2.34 [1.70, 3.20], *p* < 0.001; *I*^2^: 0%, *p* = 0.76) (Figure 3).

## 4. Discussion

This review showed that only miRNAs miR-30a, miR-30c, miR-30e, and miR-188 have been validated in human models. Meta-analysis demonstrated that miR-30a and miR-30e upregulation was associated with a higher risk of CIN.

The pathophysiology of CIN is yet to be fully elucidated; however, medullary ischemia, the formation of reactive oxygen species, and direct tubular cell toxicity might play a critical role in the pathology [15]. This is further strengthened by the fact that interventions targeting ischemia-reperfusion mechanism and suppressing free radical formations have been shown to reduce CIN incidence [16, 17]. Mature miRNAs are single-stranded RNAs that act as posttranscriptional regulators by binding mRNAs and degrading them [5]. miRNAs might be upregulated or downregulated in both acute and chronic kidney injuries [5]. A coexpression analysis demonstrated the role of miRNA-mRNA interactions in the development of contrast-induced kidney injury which affects inflammation, energy metabolism, oxidative stress reaction, cell proliferation and death, and interstitial fibrosis [10]. It is shown in Sun et al. study that the change in miRNA manifest in 4-6 hours after CM exposure, earlier than the change in serum creatinine or cystatin C and may serve as early biomarker for CIN [14].

miR-188 regulates the MAPK-JNK/p38 pathway, an important pathway of CIN [18, 19]. miR-188 has been shown to exacerbate contrast-induced apoptosis by binding to SRSF7 [12]. Overexpression of miR-188 has been shown to directly inhibit SRSF7 expression [12]. Furthermore, 3 long-noncoding RNAs (lncRNAs) LOC284581, XLOC_002253, and KCNQ1OT1 were downregulated in CIN; these lncRNAs can bind to miRNA-188 and prevent SRSF7 inhibition [12]. This mechanism shows that miR-188 may not only be an early biomarker for CIN but might also a potential drug target for CIN prevention. In animal model with diabetic kidney disease, PI3K/AKT activation via miR-188-5p can be attenuated by triptolide administration [20]. miR-21 has been shown to attenuate contrast-induced renal apoptosis by inhibiting the expression of programmed cell death protein 4 (PDCD4) in a study using HK-2 and human embryonic kidney (HEK)-293 T cells [21].

On the other hand, miR-187 has been shown to reduce acute ischemic renal podocyte injury in preclinical studies; this is achieved by inhibiting acetylcholinesterase [22]. Another study on miR-155 shows that by regulating the TCF4/Wnt/*β*-catenin pathway, acute kidney injury can be reduced [23]. Although the latter 2 studies were not on CIN, there might be an overlap in pathophysiology which involves ischemia, reperfusion, and free radical formations. Low expression of miR-30a has been associated with metastasis and poor prognosis in renal cell carcinoma (RCC) [24]. miR-30a-5p has been demonstrated to downregulate glucose-regulated protein78 (GRP78), subsequently suppressing RCC cell growth and promoting apoptosis of RCC cells as shown in a preclinical study [25]. Increased miR-30a in CIN might be related to contrast-induced apoptosis, similar to miR-188. These show that the characterization of miRNA upregulation/downregulation involved in the pathophysiology may elaborate potential agonist or antagonist for therapeutic purposes.

### 4.1. Translational Implication

miR-30a, miR-30c, miR-30e, and miR-188 are potential miRNAs that can be translated into clinical practice. miR-30a and miR-30e have been shown to be upregulated in CIN patients in meta-analysis of studies that have conducted human validation. miR-188 has been demonstrated to exacerbate kidney damage by inducing apoptosis and can be a potential drug target. While this agent may not be an ideal biomarker for early detection due to its cost, and the high prevalence of CIN, this finding provides insight for the pathophysiological importance of miRNA that serves as a basis for future research. Future research should explore on the prognostic value of these biomarkers (including progression to severe acute kidney injuries and major adverse cardiovascular events) and whether miRNA can be a target for therapy to reduce CIN.

### 4.2. Limitation

The limited amount of study is the most concerning limitation; the findings were mostly hypothesis generating. There is also a concern for publication bias, in which negative studies were less likely to be published.

## 5. Conclusion

This study highlighted the importance of certain miRNAs in CIN pathophysiology. Future researches should explore on the prognostic and therapeutic implication of miRNA in CIN.

## Figures and Tables

**Table 1:** Summary of the included studies.

Authors	Animal model	Human validation	Contrast	Control	CIN definition	Validated miRNA	Funding
Cheng 2019	Sprague-Dawley rats (250-300 g)	N/A	Iohexol	Saline	N/A	N/A	National Natural Science Foundation of China, National Science and Technology Support Program of China
Liu Y 2019	Sprague-Dawley rats (200-220 g)	N/A	Nonionic low-osmolar iodic contrast medium iopromide	Saline	N/A	N/A	National Science Foundation for Young Scientists of China, National Science Foundation of China, Science and Technology Planning Project of Guangdong Province, Guangdong Provincial Medical Research Fund Project, Guangdong Provincial People's Hospital Clinical Transformation Research Project
Liu B 2019	Sprague-Dawley rats (200-220 g)	N/A	Isotonic CM iodixanol	Saline	Increase in serum Scr or Cys C concentration of 25% above baseline within 48 to 72 h after contrast administration	N/A	National Science Foundation for Young Scientists of China, National Science Foundation of China, Science and Technology Planning Project of Guangdong Province, Guangdong Provincial Medical Research Fund Project, Guangdong Provincial Research Fund for Science and Technology, Guangdong Provincial People's Hospital Clinical Research Project
Wang 2019	Sprague-Dawley rats (300-400 g)	N/A	Nonionic monomeric low-osmolarity CM iopromide	Saline	N/A	N/A	The Natural Science Foundation of Fujian province, The Outstanding Youth Science Fund Project of No. 900 Hospital of Chinese PLA, The Health Care Project of Chinese PLA
Sun 2016	Sprague-Dawley male rats (250-300 g)	CAG/PCI patients	Nonionic, low-osmolar CM, iohexol	Saline	Absolute increase in SCr ≥ 0.3 mg/dL or relative Increase in SCr ≥ 25% or CyC ≥ 10% over baseline 24-48 hours after CM administration	miR-188, miR-30a, and miR-30e	National Natural Science Foundation of China, Shanghai Municipal Commission of Health and Family Planning, Shanghai Jiaotong University
Gutierrez-Escolano 2015	Sprague-Dawley male rats (220-300 g)	PCI patients	Type unknown	PBS	Absolute increase in SCr to ≥0.5 mg/dL or relative Increase in SCr ≥ 25% over baseline 24-48 hours after CM administration	miR-30a, miR-30c, and miR-30e	N/A

CAG: coronary angiography; CIN: contrast-induced nephropathy; PCI: percutaneous coronary intervention; miRNA: microRNA; N/A: not available/applicable.

## Data Availability

The data used to support the findings of this study are included within the article.
